# Development of Mechanical Stability in Late-Stage Embryonic Erythroid Cells: Insights From Fluorescence Imaged Micro-Deformation Studies

**DOI:** 10.3389/fphys.2021.761936

**Published:** 2022-01-10

**Authors:** Luis F. Delgadillo, Yu Shan Huang, Sami Leon, James Palis, Richard E. Waugh

**Affiliations:** ^1^Department of Biomedical Engineering, University of Rochester, Rochester, NY, United States; ^2^Department of Pediatrics, School of Medicine and Dentistry, University of Rochester, Rochester, NY, United States; ^3^Department of Biostatistics and Computational Biology, School of Medicine and Dentistry, University of Rochester, Rochester, NY, United States

**Keywords:** erythrocyte, membrane, cytoskeleton, FIMD, mechanics, stability, maturation, embryonic

## Abstract

The combined use of fluorescence labeling and micro-manipulation of red blood cells has proven to be a powerful tool for understanding and characterizing fundamental mechanisms underlying the mechanical behavior of cells. Here we used this approach to study the development of the membrane-associated cytoskeleton (MAS) in primary embryonic erythroid cells. Erythropoiesis comes in two forms in the mammalian embryo, primitive and definitive, characterized by intra- and extra-vascular maturation, respectively. Primitive erythroid precursors in the murine embryo first begin to circulate at embryonic day (E) 8.25 and mature as a semi-synchronous cohort before enucleating between E12.5 and E16.5. Previously, we determined that the major components of the MAS become localized to the membrane between E10.5 and E12.5, and that this localization is associated with an increase in membrane mechanical stability over this same period. The change in mechanical stability was reflected in the creation of MAS-free regions of the membrane at the tips of the projections formed when cells were aspirated into micropipettes. The tendency to form MAS-free regions decreases as primitive erythroid cells continue to mature through E14.5, at least 2 days after all detectable cytoskeletal components are localized to the membrane, indicating continued strengthening of membrane cohesion after membrane localization of cytoskeletal components. Here we demonstrate that the formation of MAS-free regions is the result of a mechanical failure within the MAS, and not the detachment of membrane bilayer from the MAS. Once a “hole” is formed in the MAS, the skeletal network contracts laterally along the aspirated projection to form the MAS-free region. In protein 4.1-null primitive erythroid cells, the tendency to form MAS-free regions is markedly enhanced. Of note, similar MAS-free regions were observed in maturing erythroid cells from human marrow, indicating that similar processes occur in definitive erythroid cells. We conclude that localization of cytoskeletal components to the cell membrane of mammalian erythroid cells during maturation is insufficient by itself to produce a mature MAS, but that subsequent processes are additionally required to strengthen intraskeletal interactions.

## Introduction

The red blood cell is perhaps the simplest cell in the human body. It has no nucleus, its interior consists largely of a concentrated solution of hemoglobin, and its mechanical stability resides entirely in its plasma membrane and a thin, membrane-associated cytoskeleton (MAS). Despite its simplicity, many mysteries remain about its function and structure, its maturation from erythroid precursors, and how abnormalities in its proteins and maturation process lead to hemolytic disease (Lux, 2016). The mechanical properties and physical stability of the red blood cell are paramount because of its role as a circulating corpuscle. The simplicity of the cell structure and its ready availability have contributed to a plethora of theoretical and experimental studies of the structures that account for its mechanical behavior (Skalak et al., 1973; Chien et al., 1978; Evans and Skalak, 1979; Waugh and Evans, 1979; Discher et al., 1994; Li et al., 2005; Svetina et al., 2016). Key to understanding the relationship between the structure of the MAS and cellular properties is the ability to manipulate the cell mechanically and image the resulting deformation and structural reorganizations that accompany cell deformation. The most advanced studies have combined fluorescence labeling of membrane components and controlled mechanical deformation of the cell (fluorescence imaged microdeformation, FIMD) to gain insights into how specific membrane components are re-distributed during deformation (Lee et al., 1999; Picart et al., 2000; Dahl et al., 2003; Huang et al., 2017). Here, we applied this approach to document and quantify the changes in the mechanical stability of the MAS in primary mammalian erythroid cells at different stages of maturation.

The organization of the MAS in erythrocytes has been extensively studied. It consists primarily of a network of spectrin α-β dimers that self-associate at one end to form tetramers and higher oligomers, and associate at the opposite end with junctional complexes that are organized around short filaments of actin. The spectrin network is attached to the membrane bilayer at multiple points, the most prominent being associations with band-3, an integral membrane protein that serves both as an anion transporter and the principal attachment site for the MAS and other enzymes and proteins of the cell interior (Zhang et al., 2000; Anong et al., 2009). Auxiliary proteins of the MAS and its attachment sites to the membrane have been thoroughly reviewed by others (Mohandas and An, 2006; Gautier et al., 2018). Notably, abnormalities in these proteins frequently lead to a weakening of the mechanical stability of the membrane composite and are known to result in chronic hemolytic anemias, such as hereditary spherocytosis and hereditary elliptocytosis, that can be life-threatening (Palek and Sahr, 1992). Thus, the proper assembly of a mechanically stable yet deformable MAS in red blood cells is essential for human health.

The model system we use to study progressive erythroid maturation are primary primitive erythroid cells isolated from progressive stages of mouse embryogenesis. Primitive erythroblasts initiate the onset of blood cell circulation in the mammalian embryo (McGrath et al., 2003). These cells are larger than definitive erythroid cells, which ultimately become the predominant and then exclusive cell type in the late-stage embryo and postnatal organism, respectively. Primitive erythroid cells begin circulating as immature erythroblasts that progressively mature as a semi-synchronous cohort reaching the orthochromatic stage of differentiation at E12.5 in the murine embryo (Kingsley et al., 2004). We previously determined that protein components of the MAS become localized to the membrane between E10.5—E12.5 (Huang et al., 2017). Interestingly, the mechanical stability of primitive erythroid cells continues to increase independently of enucleation status even after localization of the MAS components to the membrane (Waugh et al., 2013; Huang et al., 2017).

Here we demonstrate that the creation of MAS-free regions by mechanical deformation is the result of an isotropic rupture of the MAS, creating a hole in the MAS that propagates away from the tip of the cell projection in the micropipette, forming the MAS into an open-ended cylinder within the lumen of the pipette and a MAS-free region at the tip of the projection. We further document that the likelihood of MAS rupture increases with increasing deformation and decreases with erythroid cell maturation. Finally, we show that deficiencies in the cytoskeletal protein 4.1 also lead to increased susceptibility of the membrane to rupture compared to wild-type cells at the same stage of maturation.

## Materials and Methods

### Cell Collection

All animal procedures were approved by the University Committee on Animal Resources at the University of Rochester. ICR mice were obtained from Taconic (Germantown, NY, United States). Protein 4.1R (*Epb41*) heterozygous mice, a generous gift from John Conboy (UC Berkeley), were on a C57Bl/6 background (Shi et al., 1999). Mice were mated overnight and vaginal plugs were examined the following morning. At 10.5–14.5 days of gestation, mice were killed by CO2 inhalation, followed by cervical dislocation and embryos removed from decidual tissues in PB2 {[Dulbecco PBS (GibcoBrl, Gaithersburg, MD, United States), 0.3% BSA (Gemini Bio-Products, Sacramento, CA, United States), 0.68 mmol/L CaCl2 (Sigma-Aldrich, St Louis, MO, United States), 0.1% glucose]}. Individual embryos were transferred into 35 mm dishes with PB2 plus 12.5 μg/mL heparin, and circulating fetal blood cells were collected as previously described (Huang et al., 2017). *Epb41* embryos were genotyped using the AccuStart II Mouse Genotyping Kit (Quanta Biosciences) using primer pairs *for Neo* and *Epb41* exon 4 (Shi et al., 1999). Littermate control cells were protein 4.1R(+/+).

Human bone marrow cells were aspirated from the iliac crest of healthy donors after informed consent according to the University of Rochester’s Research Subjects Review Board. The total marrow cells were diluted 1:1 with McCoy’s 5A medium (Gibco, Grand Island, NY, United States) and layered onto Ficoll–Paque (1.077 g/ml; Pharmacia, Piscataway, NJ, United States). Mononuclear cells (MNCs) from the interface band were collected after centrifugation at 300 × *g* for 30 min at room temperature. The MNCs were washed twice in McCoy’s 5A medium and then resuspended at a cell density of 1 × 10^6^ cells/ml in filter-sterilized PBS (Gibco) supplemented with 5% v/v FBS (Gibco), 4.5 g/L D-Glucose (Sigma, St. Louis, MO, United States), 0.2 mM L–glutamine (Gibco), 50 U/ml penicillin (Gibco), and 50 μg/ml streptomycin (Gibco). Cells were labeled by incubation for 30 min at 4°C in the dark with R-phycoerythrin (PE)-conjugated antibody to glycophorin A (DAKO/Agilent, Santa Clara, CA, United States).

### Fluorescence Imaged Microdeformation

Embryonic blood cells in PB2 buffer at 300 mOsmols at room temperature were labeled with Ter-119-Alexa 488 antibody (0.5 mg/ml) for 30 min in the dark at room temperature. The cell concentration for antibody labeling was kept constant for all gestation days. After antibody labeling, the cells were suspended at high dilution in a chamber on the stage of an inverted fluorescence microscope (Nikon TE2000-E, Roper Quantem512SC digital camera, and Elements software, Nikon Instruments). Individual cells were aspirated into a micropipette at a pressure of approximately 1.47 kPa (15 cm H_2_O). This pressure was sufficient to minimize the appearance of folds in the membrane projection in the micropipette and to form the portion of the cell outside the pipette into a spherical shape. Brightfield and fluorescence images were then captured and saved for later analysis to assess cytoskeletal distribution. Formation of a membrane skeleton-free region of the lipid bilayer was observed as differences in the extension lengths of fluorescently-labeled membrane skeleton and the full cell projection observed in brightfield microscopy. Bright field images were taken using a 40× objective lens at an exposure of 60 ms with a 120 multiplier and fluorescence images were obtained with the same objective lens at an exposure of 500 ms with a 500 multiplier. After an image was taken, the cell was released. the pressure brought back to the original starting point, and a different cell was selected. The process was repeated 5–10 times using the same micropipette.

### Ghost Protocol

Transient exposure to low ionic strength buffer causes red blood cells to swell and burst, releasing cell contents and allowing large molecules outside the cell to enter (Nash and Meiselman, 1983). The resulting red cell ghosts have lost hemoglobin content and their color. Mouse primitive cells were spun down at 1,000 × *g* for 5 min. The supernatant was removed, and the cells were washed 3× at room temperature with PBS (Gibco, pH 7.4, osmolarity adjusted using distilled water to 292 mOsm). Meanwhile, 300 U of Alexa 488 phalloidin (Invitrogen) were dissolved in 1.5 mL of ethanol, and 12 μL of this solution was dried onto the bottom of an amber vial. A 50% suspension of cells (20 μL) was added to the vial and chilled to 0.0°C. 80 μL of chilled lysis buffer (7.5 mM Na_2_HPO_4_ and 7.5 mM NaH_2_PO_4_ mixed to obtain a pH of 6.0, plus a protease inhibitor (0.1 mM phenyl-methyl-sulfonyl fluoride) and incubated for 5 min at 0.0°C pH 6.0. We note that it is critical to maintain constant temperature and pH throughout the lysing and resealing steps. Buffers and cells were pre-cooled prior to mixing. 12 μL of resealing buffer [2M KCL, 21 mM MgCl_2_, 10 mM ATP, 25 mM tris (hydroxymethyl)aminomethane], 0.1 mM dithioerythritol was added and the suspension was stirred for 10 min at 0.0°C, followed by 45 min incubation at 37°C. Cells were pelleted at 1,000 × *g* for 4 min, and after supernatant removal, cells were washed three times with PBS (292 mOsm) and two times with PBS (210 mOsm) with 5% fetal bovine serum (FBS). After washing, the cells were suspended in PBS (210 mOsm) with 5% FBS for measurement.

### Statistics

Analysis of the FIMD images was carried out using measurements of the brightfield and fluorescence images. The percentages of cells that showed skeleton-free regions for a given gestation day and extension ratio, were compared. Statistical significance was assessed using a logistic regression that models the probability of MAS failure given the covariates cell extension (*L*_*e*_/*R*_*p*_) and gestation day. Statistical differences were assessed for different days compared against the data for cells from E14.5 (*p* < 0.05). Logistic regression was also performed comparing results for protein 4.1R-null cells with littermate controls that were homozygous for 4.1R expression (WT).

## Results

### Observations of the Formation of Membrane-Associated Cytoskeleton-Free Membrane Regions

**FIGURE 1:**
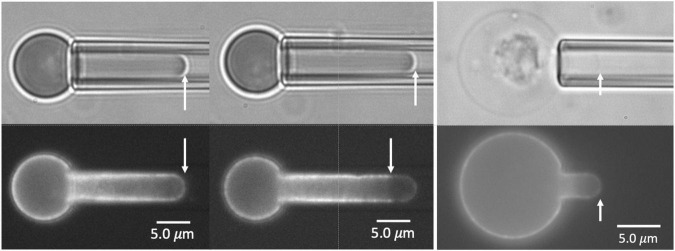
Images of E13.5 primitive erythroid cells aspirated into a micropipette. Two pairs of images for each of three cells are shown, brightfield at the top, fluorescence at the bottom. In the left and center panels, the fluorescent label is TER-119. In the right panels, the label is Alexa-488 phalloidin. The cell at the left shows bright edge-labeling around the entire projection of the cell in the micropipette, including the tip, indicating an intact MAS. The cell in the center shows fluorescent labeling that stops approximately 3.0 μm from the tip, indicating rupture of the MAS and formation of a MAS-free region at the tip of the projection. The rightmost cell is a ghost cell with an intact MAS from an E14.5 embryo. Scale bars as shown.

The Ter-119 antibody associates with glycophorin A and increases its association with the MAS, making it a reliable indicator of MAS localization (Lee et al., 2004). Inspection of FIMD images of aspirated primitive erythroid cells revealed that in a significant fraction of aspirated cells, the fluorescence distribution did not fully extend to the tip of the micropipette where the bright field image shows the cell to be present (Figure 1). These differences in the extension lengths of fluorescently labeled MAS and the full cell projection observed in brightfield demonstrate the formation of MAS-free regions of the membrane. Measurements of fluorescence distribution using FIMD have enabled us to determine the frequency with which such regions form and how this frequency changes with both the magnitude of the deformation and the maturity of the cells.

**FIGURE 2:**
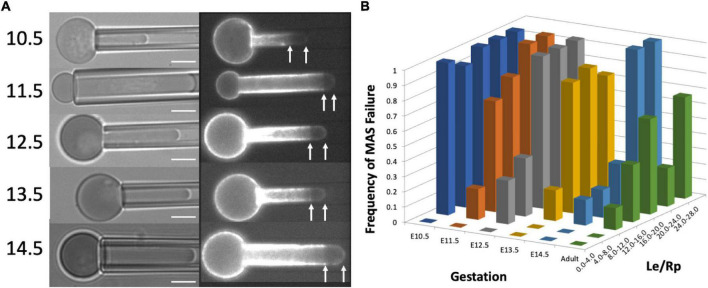
**(A)** Images of cells from each gestation day showing failure of the MAS. Scale bar = 5.0 μm. **(B)** The dependence of the frequency of MAS failure on projection length (*L*_*e*_/*R*_*p*_) and cell maturation (gestation day). Each column represents the fraction of cells observed to have MAS-free regions for the range of *L*_*e*_/*R*_*p*_ ratios shown on the right-hand axis. Approximately 50 cells were measured for each different gestation day (except E13.5, for which there were 28 cells measured). The number of cells represented by each column depended on the distribution of projection lengths for the cell population and ranged from 1 to 27. Based on logistic regression to the entire data set (without binning), all days were significantly different from E14.5, except for the adult definitive cells, which were not significantly different from primitive cells on E14.5.

Membrane-associated cytoskeleton-free regions in micropipette-aspirated cells appeared at all stages of maturation tested (Figure 2A). The frequency with which these regions formed decreased with increasing maturation and increased with the magnitude of deformation. Cell maturation was gauged by the gestation day at which the cells were harvested, (E10.5 through E14.5), since primitive erythroid cells differentiate as a semi-synchronous cohort, reaching the basophilic erythroblast stage at E10.5 and the orthochromatic erythroblast stage by E12.5-E13.5 (Kingsley et al., 2004, 2013). The magnitude of deformation was gauged by measuring the ratio of the length of the extended projection of the cell inside the micropipette *L*_*e*_ to the radius of the pipette *R*_*p*_. This ratio, *L*_*e*_/*R*_*p*_, provides an indication of the maximum mechanical extensions of the cell membrane (Evans and Skalak, 1979). At E10.5, nearly all the cells tested exhibited the formation of MAS-free regions, regardless of the magnitude of deformation. As the cells matured (at later days of gestation), larger and larger deformations were required in order to observe MAS-free regions. To characterize this dependence, 40–50 cells were tested from multiple embryos at each day of gestation. The data were binned according to the measured ratio *L*_*e*_/*R*_*p*_, and the fraction of cells exhibiting MAS-free regions was calculated for each group (Figure 2B). Based on logistic regression, the likelihood of failure at a given extension was significantly higher for all days relative to E14.5, except the adult definitive cells, which did not show a statistical difference in failure rate compared to E14.5.

**FIGURE 3:**
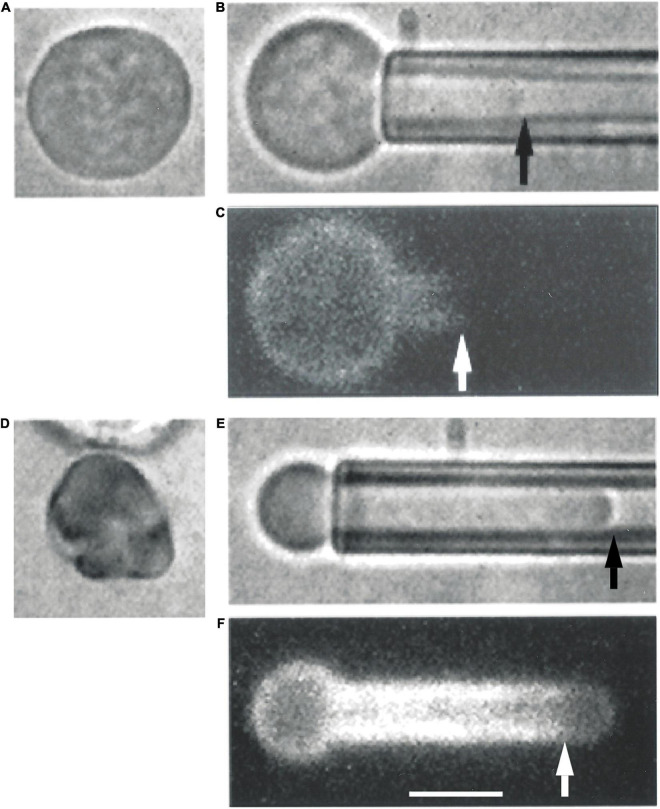
Erythroid cells from adult human marrow also exhibit the formation of MAS-free regions. Two cells prior to aspiration are depicted in panels (**A**,**D**). Panel **(A)** shows an erythroid precursor identified by a darker color in blue illumination due to the presence of Hb in the cell. The cell in panel **(D)** appears to be a marrow reticulocyte, based on its lobular appearance and lack of a nucleus. When the cell in panel **(A)** was aspirated into a micropipette, the distribution of fluorescence resembled that of primitive mouse cells from E10.5 [panels **(B,C)**]. When the cell in panel **(D)** was aspirated, the fluorescence distribution resembled that of primitive erythroid cells obtained at E14.5 [panels **(E,F)**]. Scale bar is 5.0 μm for all images.

Similar phenomena were observed in immature erythroid cells harvested from adult human marrow (Figure 3). Maturing erythroblasts obtained directly from marrow can be identified by their high contrast in blue illumination (reflecting hemoglobin content), but cells at all stages of maturation are represented. Therefore, we must rely on morphology to estimate the level of cell maturity. Two examples of human marrow erythroid cells subjected to FIMD are shown in Figure 3. One cell appears to be similar to erythroid cells found in mouse embryos at E10.5 or before, and the other appears to be a multi-lobulated marrow reticulocyte, probably similar to primitive erythroid cells found in mouse embryos at E14.5. In both of these example cells, MAS-free regions are observed, demonstrating that this phenomenon also occurs in cells maturing in adult human marrow.

### Deficiencies in the Cytoskeletal Protein 4.1 Enhance the Formation of Membrane-Associated Cytoskeleton-Free Regions

**FIGURE 4:**
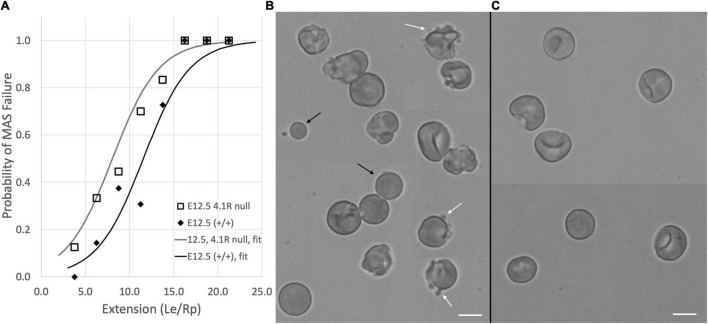
**(A)** Protein 4.1R deficiency resulted in less stable membranes as indicated by a decrease in the deformation required to produce MAS-free regions. Data for fort-nine E12.5, 4.1R null cells and forty-seven cells from (+/+) littermate controls are shown. Each of the five middle bins contains data from six to thirteen cells. Only a few cells could be found for the lowest and highest ranges. Solid curves show the logistic regression for each group. Differences between the two groups were statistically significant. **(B)** The instability of the 4.1R-null cells is also evident in the appearance of blebs (white arrows) and spherical cell fragments (black arrows) in E12.5 protein 4.1 null cells. **(C)** Similar structures are not observed in littermate controls. Panels **(B,C)** are composites of cell images collected from different parts of the image field to increase the number of cells that could fit into a publication-sized image. Bars = 10 μm.

The erythroid-specific isoform of protein 4.1 (protein 4.1R) stabilizes the interaction between spectrin and junctional complexes within the MAS (Ungewickell et al., 1979). To explore the consequences of protein 4.1 deficiency during erythroid maturation, we examined primitive erythroblasts from protein 4.1R knockout mice. Previously we have reported that the recruitment of MAS proteins to the cell membrane between E10.5 and E12.5 appears to occur normally in these mice, but that the membranes of the 4.1R-null primitive erythroid cells at E12.5 were mechanically less stable (Huang et al., 2017). Here we further document the mechanical instability of these cells and the spherocytic phenotype of the adult red blood cells in these mice. Protein 4.1R-null cells showed a greater tendency to form MAS-free regions than wild type cells when deformed into micropipettes. This is illustrated in Figure 4, where we plot the fraction of E12.5 primitive erythroid cells exhibiting MAS-free regions for different ranges of deformation (*L*_*e*_/*R*_*p*_). Note that for the littermate controls (resulting from the mating of mice that are heterozygous for the missing protein 4.1R gene) the increase in the fraction of cells exhibiting MAS-free regions is shifted toward higher values of *L*_*e*_/*R*_*p*_. Based on logistic regression analysis, this difference is statistically significant, indicating that less deformation is required to produce MAS-free regions in the 4.1R-null cells, consistent with a less-stable MAS. The *L*_*e*_/*R*_*p*_ ratio needed to generate a 50% failure rate was approximately 8.0 for the protein 4.1R-null cells and approximately 12.0 for the littermate controls. There was no significant difference in stability measures between the normal protein 4.R littermate control cells (on a C57Bl/6 background) compared to wild type E12.5 erythroblasts (ICR mice). Cells at E10.5 do not express the erythroid-specific splice variant of protein 4.1 (4.1R) and lack many of the key components of the erythroid-specific MAS (e.g., band-3, protein 4.2, β-actin) at the cell membrane (Huang et al., 2017). At this stage, 4.1R-null and 4.1R-positive cells behaved similarly, all cells showing Ter-119-free regions for even small deformations.

Membrane instability was also evident in the cell morphology (Figures 4B,C). Protein 4.1R-null cells exhibited blebbing behavior, and spherical cell fragments could be observed in the cell suspension. The membrane instability resulted in a more spherical phenotype for the protein 4.1R-null cells even at E12.5. This was quantified by calculating the area *A* and volume *V* of the pipette-aspirated cells using measurements of the radius of the spherical portion of the cell outside the pipette *R*_*s*_, the length of the projection inside the pipette *L*_*e*_, and the pipette radius *R*_*p*_:


A=2⁢π⁢Rs⁢[Rs+Rs2-Rp2]+2⁢π⁢Rp⁢Le

V=2⁢π3⁢[Rs3+(Rs2+Rp22)⁢Rs2-Rp2+Rp3]+π⁢Rp2⁢(Le-Rp)

How spherical a cell is can be expressed in terms of the “sphericity,” a dimensionless ratio of the volume to the 2/3 power divided by the cell area:


$$S=\left(\frac{4\,\pi }{{\left(4\,\pi /3\right)}^{2/3}}\right)\,\frac{V^{2/3}}{A}$$

**FIGURE 5:**
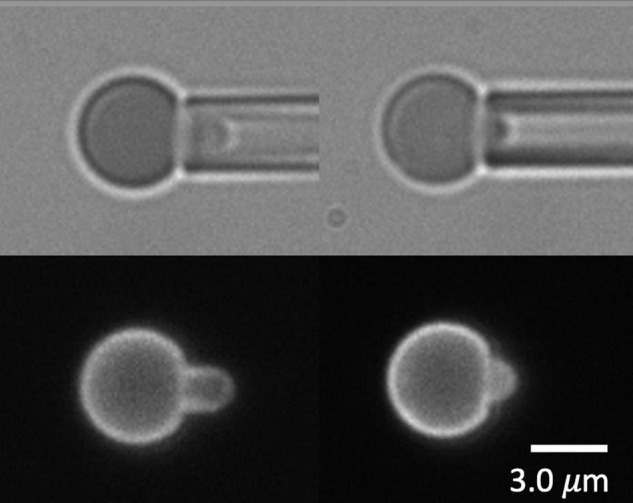
Erythrocytes from an adult protein 4.1R-null mouse shown in brightfield **(top)** and labeled with Ter-119 **(bottom)**. Cells were too spherical to form projections long enough to cause failure of the MAS.

A perfect sphere has a sphericity of 1.0. The sphericity for E12.5 4.1R null cells (mean ± standard deviation) was 0.86 ± 0.06, *n* = 49, and the sphericity for the WT cells of the littermate controls was 0.78 ± 0.09, *n* = 37, a statistically significant difference (Student’s *t*-test). The sphericity of the 4.1R null cells remained high throughout maturation, making it difficult to make meaningful comparisons between the null cells and their littermate controls beyond E12.5 because the magnitude of the deformation was so different between the two cell types. By the time the null cells reached maturity, hardly any membrane projection into the pipette could be achieved (Figure 5).

### Mechanism of Failure: Detachment From Bilayer or Lateral Dissociation?

**FIGURE 6:**
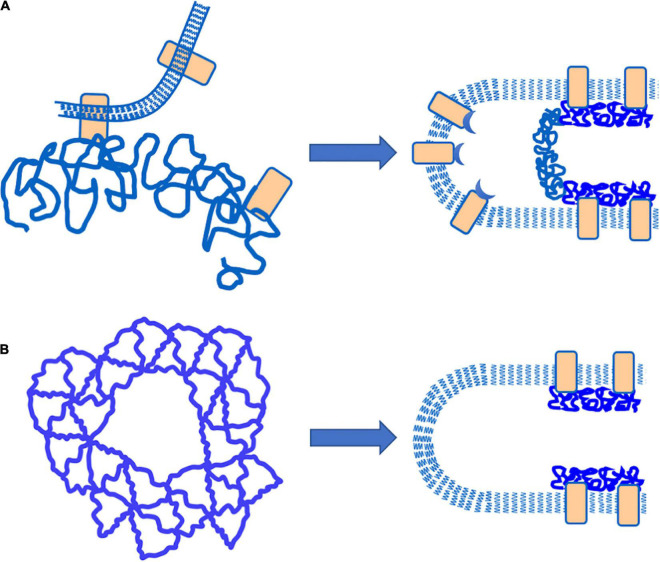
Schematic illustration of different failure mechanisms that would result in the creation of MAC-free regions of the plasma membrane. **(A)** “Vertical” failure involves detachment of the MAC from the plasma membrane by breaking bonds between the MAC and integral proteins embedded in the plasma membrane. In this case, a “septum” of intact MAC should exist across the lumen of the cell projection. **(B)** “Horizontal” failure involves tearing apart the lateral connections within the MAC itself. In this case, an open-ended cylinder of MAC is expected to form along the walls of the cell projection.

There are two basic ways in which MAS-free regions of the plasma membrane can be formed. One is by the “vertical” detachment of the MAS from the membrane bilayer, and the other is by a lateral dissociation of proteins forming MAS network, creating a “hole” that propagates laterally along the length of the cell projection. Interpretation of the separation experiments depends critically on which of these two mechanisms is occurring. A cartoon representation of the two different mechanisms is shown in Figure 6 along with the consequent organization of the MAS and the bilayer membrane after failure. Note that for a “vertical” failure, the MAS network remains intact, forming a flat septum of networked proteins across the lumen of the cell projection. In the case of lateral (“horizontal”) failure, the lumen of the cell projection remains open as the MAS contracts along the cylindrical portion of the membrane projection. Labeling extracellular proteins on the surface of the membrane, such as TER-119 labeling, does not permit us to distinguish between these two mechanisms, but intracellular labeling of the MAS itself does.

**FIGURE 7:**
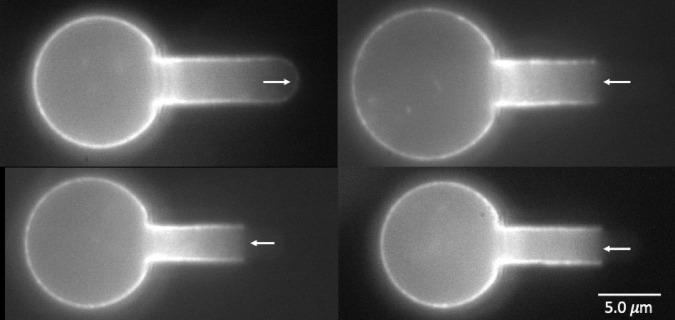
Images of four wild type primitive erythrocyte ghosts labeled with Alexa 488 phalloidin. Top left, E15.5, intact cell; lower left E14.5, top cell at right, E15.5, bottom cell right, E13.5. Right pointing arrow (top left) indicates edge brightness in the pipette lumen at the intact cell tip, left-pointing arrows show lack of edge brightness in the pipette lumen for the three cells exhibiting MAS-free regions.

To label the MAS intracellularly, red cell “ghosts” were made from primitive erythroid cells, enabling the incorporation of Alexa-488 phalloidin into the interior of the cell to label the actin portion of the MAS. Examples of cells with fluorescently labeled actin are shown in Figure 7. One intact MAS example and three ruptured MAS examples are shown. Note the absence of “edge-bright” fluorescence in the lumen of the pipette, except at the tip of the intact example. These images provide clear evidence that the failure mechanism we are observing represents a lateral tearing (rupture) of the MAS and the formation of an open-ended cylindrical sleeve of MAS inside the cell projection into the micropipette. This further reveals that our approach assesses the strength of lateral attachments within the MAS and how this strength increases with maturation.

### Dimple Formation Reveals a Contractile Tension at the Edge of the Torn Membrane-Associated Cytoskeleton

**FIGURE 8:**
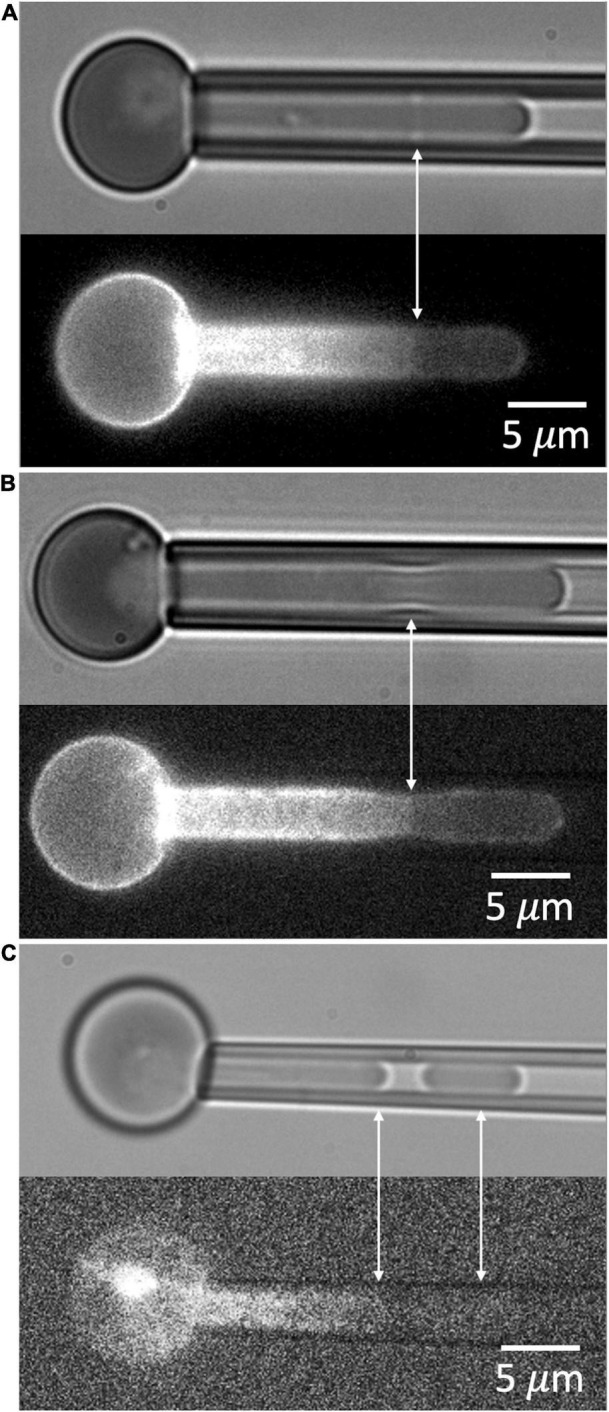
**(A)** An example of the dimple formed at the edge of the ruptured MAS when aspiration pressure is reduced. **(B)** A second example showing necking at the edge of the MAS. **(C)** An example of the formation of a MAS-free fragment. Cells in panels (**A**,**B**) are from an E14.5 embryo, and the cell in panel **(C)** was from an E13.5 embryo.

**FIGURE 9:**
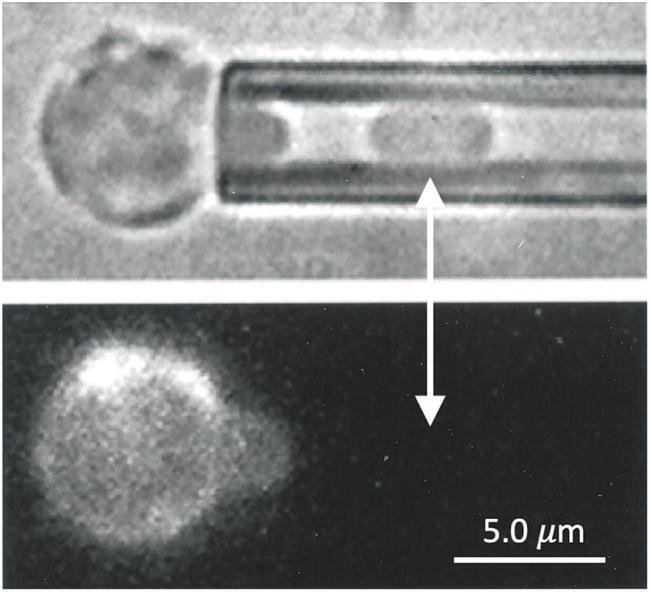
Primary human erythroid precursors also exhibit the formation of MAS-free fragments when aspirated into a micropipette. The cell fragment, clearly visible in the **(top panel)**, shows no evidence of glycophorin A labeling **(lower panel)**.

When a red blood cell is fully aspirated into a micropipette, a pressure is generated inside the cell that is greater than both the aspiration pressure in the micropipette and the pressure in the suspending medium outside the cell. This pressure acts to press the membrane against the inner wall of the pipette lumen and maintain its cylindrical geometry. In cases where the MAS has been torn and forms a truncated cylinder inside the cell projection, an indentation can form at the edge of the torn skeleton when the aspiration pressure, and the corresponding pressure inside the cell, are reduced. This phenomenon is illustrated in Figure 8. Images showing the dimple formation in brightfield microscopy and the corresponding distribution of fluorescence in FIMD are shown in Figure 8A. This phenomenon indicates that despite having ruptured, the MAS retains some elastic cohesion at its edge that would contribute to cell fragmentation leading to the loss of MAS-free regions of the cell membrane. In Figure 8B we show an example of a projection that is “necking” down at the edge of the MAS, a step prior to cell fragmentation. An example showing complete fragmentation of a maturing primitive erythroid cell is shown in Figure 8C. Note that TER-119 labeling of the MAS is absent from the separated fragment indicating that cell fragments formed in this way lack MAS components. Formation of MAS-free fragments are also observed in primary erythroid cells from human marrow (See Figure 9).

## Discussion

### The Power of Fluorescence Imaged Microdeformation

The combination of fluorescence labeling of cell structural components and applied micro-deformation is a powerful approach for understanding the structural mechanisms leading to cell behavior in response to applied forces. The application of this experimental approach to adult human red blood cells revealed that the local density of the MAS changes when cells are deformed (Discher et al., 1994). This was significant because many theoretical treatments of red blood cell mechanical behavior had previously made the assumption that the density of the MAS was uniform in deformation (Evans and Skalak, 1979). The FIMD observations supported the development of more complex theoretical descriptions that account for changes in local MAS density (Dao et al., 2006; Peng et al., 2010; Svetina et al., 2016; Feng et al., 2020). In this report, we document an important mechanism of MAS failure in primary primitive erythroid cells from mice, namely the lateral separation of MAS components to form a hole in the MAS. As was observed for the energy cost of tether formation in prior studies (Waugh et al., 2001, 2013), the tendency of the MAS to undergo lateral failure decreases as the cells mature in the embryo, even days after the MAS protein components become localized to the membrane. These observations lead to the conclusion that localization of the MAS components to the membrane is not in itself sufficient to allow full MAS assembly, but that additional events are required after localization to further strengthen the inter-protein connections with the MAS.

Whenever cell labels are used one should consider the possibility that the label itself could have effects on cell behavior. This is relevant in the current context. Lee et al. (2004) have shown that labeling mouse erythrocytes with TER119 increases their mechanical stiffness (Lee et al., 2004), consistent with the observation that antibodies against human glycophorin A increase the stiffness of human red blood cells (Knowles et al., 1994). It is also conceivable that the use of phalloidin as a label for the actin junctional complexes could also affect the stability of junctional complexes if the label competes for binding with native proteins in the MAS, although this has never been documented, to our knowledge. It is worth noting, however, that in the limited number of phalloidin labeled ghosts of E14.5 embryos that we tested, a high fraction of cells exhibited MAS-free regions for *L*_*e*_/*R*_*p*_ ratios > 8.0, whereas in intact cells labeled with TER119, the extension at which 50% of cells form MAS-free regions was approximately 18.7. This discrepancy could be the result of alterations to the MAS during the ghost preparation process, an effect of the phalloidin, or an effect of the TER119. The fact that both labeling approaches show consistent trends lends confidence that our general conclusions are valid, and that the behavior of unlabeled, intact cells likely falls in the range between the two labeling approaches.

### Membrane-Associated Cytoskeleton Development During Erythroid Maturation

The development of a functional MAS during erythroid maturation is fundamentally important for health. Much of the research in this area has focused on the expression and localization of the principal proteins of the MAS (Minetti et al., 2018). The challenge in most of these studies is the ability to define specific stages of maturation so that the proper sequence of events can be ascertained. Early studies relied on non-mammalian models, or erythroleukemia cells in order to observe the sequencing of protein synthesis (Cox et al., 1987; An et al., 2014). With the advent of flow cytometric methods to assist with lineage definition and cellular staging (Hu et al., 2013), and approaches to detect both messenger RNA and proteins in small quantities (An et al., 2014; Gautier et al., 2018), more recent studies have been able to refine our understanding of the synthesis of MAS components and their assembly in primary erythroid cells at progressive stages of maturation, both mouse and human. The principal MAS proteins, with the exception of actin, progressively increase as a percentage of total protein during maturation from proerythroblasts through the orthochromatic erythroblast stages (Hu et al., 2013). Our model system using primitive erythroid cells that mature as a semi-synchronous cohort in mouse embryos provides an alternate approach for evaluating MAS synthesis and assembly in a primary mammalian system. Using this approach, we have previously demonstrated that the principal protein components of the MAS are significantly synthesized as early as the proerythroblast stage (E10.5) and that their presence as a percentage of total protein continues to increase through the orthochromatic stage (E12.5) (Huang et al., 2017). The localization of MAS components at the membrane only occurs after the proerythroblast stage, timing that coincides with the beginning of the erythroid-specific splicing of protein 4.1 mRNA in a narrow time interval between E10.5 and E11.5 (Huang et al., 2017).

These approaches have added considerably to our understanding of protein and gene expression during terminal erythroid differentiation, but studies documenting the functional development of the MAS during maturation, particularly those that focus in the critically important development of mechanical stability during maturation, have remained a challenge. In a previous report, we documented the changes in surface area and volume, resistance to deformation, and the strength of the bilayer-skeletal association in maturing primitive erythroid cells from E12.5 through E16.5 (Waugh et al., 2013). While the resistance to membrane deformation did not change over this period, the surface-to-volume ratio of the cell increased, thereby increasing cellular deformability, and the strength of association between the MAS and the membrane bilayer also increased. This latter finding points to a continued increase in mechanical stability of cells in the late stages of erythropoiesis long after the principal protein components are localized to the membrane, a conclusion that is further supported in the current study.

It is interesting to speculate about what additional changes in the MAS proteins at the red cell surface might contribute to the continued strengthening of the MAS after E12.5. On plausible mechanism might be changes in the phosphorylation state of key MAS proteins. It has long been known that many MAS proteins can be phosphorylated and de-phosphorylated (Boivin, 1988). Of relevance to the current topic, increased phosphorylation of both protein 4.1R and β-spectrin lead to decreases in the strength of protein-protein interactions (Eder et al., 1986; Subrahmanyam et al., 1991; Gauthier et al., 2011) as well as the mechanical stability of the intact MAS *in situ* (Manno et al., 1995, 2005). Thus, one likely mechanism for the increased mechanical stability we have observed is the progressive dephosphorylation of one or both of these key MAS proteins.

### Mechanisms of Membrane-Associated Cytoskeleton Mechanical Failure and the Formation of Cell Fragments

The membrane composite (bilayer plus MAS) can fail mechanically in multiple ways. Protein-protein interactions within the red cell membrane have been classified as being “vertical” (between the MAS and the bilayer) or “horizontal” (within the MAS) (Tse and Lux, 1999). Failure of horizontal associations typically leads to fragmentation of the cell, while failure of vertical associations typically leads to bilayer detachment. As is evident from the current results, the process can be more complex than this simple picture. The lack of edge fluorescence in aspirated cells (Figure 7) shows that the measurements here involve a “horizontal” failure of the MAS, but this failure is also associated with the detachment of MAS-free bilayer from the cell (Figures 8, 9).

Given that the mechanisms underlying membrane stability are diverse, it follows that assessment of the mechanical stability of the membrane should involve multiple approaches, and that different testing methods may reveal different underlying mechanisms by which cells maintain (or lose) cohesion. In previous work, we used membrane tether formation to evaluate the strength of the association between the MAS and the membrane bilayer (Waugh et al., 2013). Tethers are thin, cylindrical strands of membrane bilayer pulled from red blood cell surfaces. They do not contain MAS (Butler et al., 2008), and therefore, the force required to form a tether provides a direct measure of the energy cost of creating an MAS-free region of membrane. This energy is manifested in the present study by the fact that after lateral rupture, the MAS cylinder inside the cell projection does not collapse to zero length, but is maintained at finite length by the energy cost of creating additional MAS-free area. Based on tether formation measurements, this energy is approximately 78 μJ/m^2^ for mature human red blood cells, which is substantially higher than for human marrow reticulocytes (19 μJ/m^2^) (Waugh et al., 2001). Similar increases were observed for maturing primitive mouse erythroid cells, the energy of association increasing from 30 μJ/m^2^ at E12.5 to 53 μJ/m^2^ at E16.5 (Waugh et al., 2013). The physical basis for this energy has yet to be ascertained. Tether formation does not require the breakage of protein-protein interactions between the MAS and the bilayer because the fluid-like membrane can simply flow between the integral membrane “posts” that are attached to the intact MAS. One plausible mechanism for this energy cost is the concept of an “osmotic tension” generated by the different mole fractions of lipid in the membrane region that is associated with the membrane bilayer and in the region that is not (Waugh and Bauserman, 1995).

Caution should be exercised in extending the present findings to interpret membrane fragmentation events and extracellular vesicle (EV) formation from red cells in general. The fact that the MAS-free regions produced in our experiments can be pulled away to form fragments does not mean that all cell fragments are likely to be free of MAS components. On this point, it is relevant that labeling GPA with TER119 increases GPA association with the MAS (Lee et al., 2004), and this will likely artificially prevent the appearance of GPA in the detached vesicles in our experiments. Indeed, a proteomic study of erythrocyte-derived microvesicles from human red cells used GPA to identify RBC-derived microvesicles from plasma. Interestingly, and in contrast to some early reports (Lutz et al., 1977), vesicles isolated in this way do contain some cytoskeletal proteins (Bosman et al., 2012), although at lower proportions than in intact cells. Vorselen et al. (2018) have also found evidence of MAS proteins in EVs both from normal donors and patients with hereditary spherocytosis. It should be kept in mind that EVs can arise from a variety of mechanisms, and that these will likely lead to significant heterogeneity in composition and properties. It is also the case that methods used to isolate and identify EVs will likely lead to the preparation of different populations of EV’s reflecting contributions from different types of EVs and different levels of contamination (Hebbel and Key, 2016; Lapping-Carr et al., 2020).

### Consequences of Protein 4.1R Deficiency

Our observation that protein 4.1 deficiency leads to an increased tendency to form MAS-free regions is expected given the long-standing understanding that protein 4.1 serves to strengthen the association between spectrin and actin (Ungewickell et al., 1979), and is a key component of the junctional complexes within the MAS. While it is tempting to argue based on the absence of membrane strengthening we see in the absence of protein 4.1 that protein 4.1 may play a role in the strengthening of the MAS during maturation. However, a network is only as strong as its weakest link, and it is at least as likely that in the presence of protein 4.1, other associations (such as spectrin-spectrin interactions) are the first to fail, and that it is the strengthening of these associations that leads to a more stable MAS overall. Indeed, there is evidence in the literature about the lability of spectrin-spectrin associations in response to surface deformation (An et al., 2002), and it is known that deficiencies in this association can lead to hemolytic anemia in the form of hereditary elliptocytosis (Nicolas et al., 1998). In human patients, abnormalities in protein 4.1 (as is the case for abnormalities affecting spectrin self-association) typically lead to elliptocytosis (Palek and Sahr, 1992). Complete 4.1 deficiencies in human patients are rare, but in the few cases that have been identified, the phenotypic presentation includes characteristics of elliptocytosis and spherocytosis (Feo et al., 1980; Baklouti et al., 2011). A new insight provided by the present study is that the spherocytic phenotype is detectable as early as E12.5 in primitive erythroid cells, a stage equivalent to orthochromatic erythroblasts. It is unknown whether protein 4.1 deficient cells in the marrows of human patients exhibit similar characteristics. The precise nature of the lateral associations within the MAS that are being broken and the associated biochemical changes that serve to increase the strength of those associations during maturation will be the subject of future studies.

## Data Availability Statement

The raw data supporting the conclusions of this article will be made available by the authors, without undue reservation.

## Ethics Statement

The studies involving human participants were reviewed and approved by Institutional Review Board, Office of Human Subjects Protection, University of Rochester. The patients/participants provided their written informed consent to participate in this study. The animal study was reviewed and approved by University Committee on Animal Resources, University of Rochester.

## Author Contributions

LD performed all of the experiments involving FIMD on primitive erythroid cells except for the actin-labeled ghost experiments and portions of the materials and methods section and the results section were adapted from his Master’s Thesis at the University of Rochester. YH provided most of the primitive erythroid cells used in these studies and performed genotyping of the Epb41 mouse embryos. SL provided statistics expertise and performed statistical analysis of the failure data for Figures 2B, 4A. JP provided oversight to YH as her doctoral advisor and helped co-write the manuscript with RW. RW wrote the main draft of the manuscript and oversaw all FIMD experiments on both embryonic mouse and adult human erythroid cells. All authors contributed to the article and approved the submitted version.

## Conflict of Interest

The authors declare that the research was conducted in the absence of any commercial or financial relationships that could be construed as a potential conflict of interest.

## Publisher’s Note

All claims expressed in this article are solely those of the authors and do not necessarily represent those of their affiliated organizations, or those of the publisher, the editors and the reviewers. Any product that may be evaluated in this article, or claim that may be made by its manufacturer, is not guaranteed or endorsed by the publisher.
